# Quality of discharge summaries prepared by first year internal medicine residents

**DOI:** 10.1186/1472-6920-12-77

**Published:** 2012-08-15

**Authors:** Kimberly Legault, Jacqueline Ostro, Zahira Khalid, Parveen Wasi, John J You

**Affiliations:** 1 Department of Medicine, McMaster University, 25 Charlton Ave. East, Suite 708, Hamilton, ON L8P 3B2, Canada; 2 Department of Medicine, Internal Medicine Residency Office, McMaster University, HSC-3W10A-C, 1200 Main St. West, Hamilton, ON L8N 3Z5, Canada; 3 Department of Medicine, McMaster University, Hamilton, Canada; 4 Departments of Medicine, and of Oncology, Juravinski Hospital and Cancer Centre, McMaster University, B3:169D, 711 Concession St, Hamilton, ON L8V 1C3, Canada; 5 Departments of Medicine, and of Clinical Epidemiology & Biostatistics, McMaster University, 1200 Main Street West, HSC-3V51B, Hamilton, ON L8N 3Z5, Canada

## Abstract

**Background:**

Patients are particularly susceptible to medical error during transitions from inpatient to outpatient care. We evaluated discharge summaries produced by incoming postgraduate year 1 (PGY-1) internal medicine residents for their completeness, accuracy, and relevance to family physicians.

**Methods:**

Consecutive discharge summaries prepared by PGY-1 residents for patients discharged from internal medicine wards were retrospectively evaluated by two independent reviewers for presence and accuracy of essential domains described by the Joint Commission for Hospital Accreditation. Family physicians rated the relevance of a separate sample of discharge summaries on domains that family physicians deemed important in previous studies.

**Results:**

Ninety discharge summaries were assessed for completeness and accuracy. Most items were completely reported with a given item missing in 5% of summaries or fewer, with the exception of the reason for medication changes, which was missing in 15.9% of summaries. Discharge medication lists, medication changes, and the reason for medication changes—when present—were inaccurate in 35.7%, 29.5%, and 37.7% of summaries, respectively. Twenty-one family physicians reviewed 68 discharge summaries. Communication of follow-up plans for further investigations was the most frequently identified area for improvement with 27.7% of summaries rated as insufficient.

**Conclusions:**

This study found that medication details were frequently omitted or inaccurate, and that family physicians identified lack of clarity about follow-up plans regarding further investigations and visits to other consultants as the areas requiring the most improvement. Our findings will aid in the development of educational interventions for residents.

## Background

Patients are particularly susceptible to medical error during transitions from inpatient to outpatient care. Recent work has shown low levels of both provider and information continuity after discharge from hospital [1]. However, a complete, accurate, and timely discharge summary can communicate important information back to the primary care physician, prevent adverse events, and reduce readmissions to hospital [2]. Key elements of a discharge summary include the identification of unresolved medical issues at the time of discharge, test results requiring follow-up, and the presence of an accurate discharge medication list. These items, when missing, can have a negative impact on patient care and could affect health outcomes [3,4]. Indeed, discrepancies between medication lists across different settings (e.g., pre-hospital, hospital admission, and post-discharge) are common [5]. Since adverse drug events after hospital discharge can lead to significant morbidity and mortality but are often preventable [6,7], improvement in medication reconciliation has become an important priority within the patient safety movement [8].

Although no uniform, validated discharge summary format currently exists, the Joint Commission on Hospital Accreditation has recommended specific items that should be included in all discharge summaries [9]. These include discharge diagnosis, discharge medication list, changes to medications, clinical course in hospital, results of relevant investigations, and follow-up instructions for the outpatient physician regarding follow-up care. However, several studies evaluating the completeness of discharge summaries have found that such information is often lacking [10,11]. For instance, a systematic review by Kripalani et al. found that the primary diagnosis, test results, discharge medications and follow-up plans were absent or incomplete in 18%, 38%, 21%, and 14% of discharge summaries respectively [11]. While these data provide useful insights regarding the completeness of discharge summaries, few studies have assessed the accuracy of the information contained within discharge summaries [12]. Moreover, very little is known about family physicians’ satisfaction with discharge summaries they receive [13].

In teaching hospitals, residents are largely responsible for completing discharge summaries. Anecdotally, there appears to be little formal teaching about discharge summaries in the curricula of most medical schools and residency programs, and many residency programs do not evaluate the quality of residents’ discharge summaries. To inform the design of an educational intervention for incoming first year internal medicine residents regarding the production of effective discharge summaries, we evaluated the completeness and accuracy of discharge summaries produced by incoming postgraduate year 1 (PGY-1) internal medicine residents at our institution, and assessed the relevance of the discharge summaries to the family physicians that receive them. We intentionally targeted novice clinicians in this study as our intent was to perform a baseline needs assessment to inform future efforts on teaching PGY-1's how to prepare discharge summaries.

## Methods

### Assessment of the completeness and accuracy of PGY-1 discharge summaries

The charts of patients consecutively discharged from the internal medicine Clinical Teaching Units (CTUs) at each of three university affiliated teaching hospitals at our institution (McMaster University Medical Centre, Hamilton General Hospital, and St. Joseph’s Hospital) between July 1, 2009 and August 31, 2009 were reviewed for eligibility. The target sample was 30 charts from each site, for a total of 90 charts. Charts were eligible for inclusion if the discharge summary for that admission was dictated by an internal medicine PGY-1 resident during their first two month CTU rotation. (At our institution, incoming internal medicine residents begin their PGY-1 year on July 1^st^ and typically begin on a CTU rotation.) Charts were excluded if the patient was discharged to a destination different than the one from which they were admitted (e.g., discharged to a new placement at a long term care facility, transferred from the CTU to a different hospital ward, or transferred to a rehabilitation or palliative care unit).

Data were abstracted independently in duplicate by pairs of investigators regarding the presence of the following essential domains described by the Joint Commission for Hospital Accreditation: discharge diagnosis, course in hospital, discharge medication list including changed medications with rationale for changes, investigations, follow-up plan, and an overall assessment of length and accuracy (see Additional file 1). The investigators recorded whether these items were present, and, when present, assessed the accuracy of these items compared to the patient chart as a reference standard. Discrepancies between the investigators' assessments were resolved by consensus.

### Assessing the relevance of discharge summaries to family physicians

In the second part, a convenience sample of family physicians, after providing written, informed consent, reviewed a separate sample of PGY-1 internal medicine residents’ discharge summaries regarding their own patients discharged from the CTU. The family physicians were asked to rate the discharge summaries on domains that family physicians had deemed important in previous studies, including discharge diagnosis, course in hospital, and follow-up plan for medications, investigations, and specialist care (see Additional file 2). The domains assessed in the chart review component of our study (assessment of completeness and accuracy) and the family physician survey were similar. However, some information (i.e., accuracy of discharge medication list) was only assessed in the chart review component of the study since the family physicians did not have access to the hospital chart and therefore would not have been able to make an accurate evaluation of this aspect of the discharge summary. Study personnel followed up with the physicians with an email reminder and in person to maximize survey response rates.

### Statistical analysis

For the first part of the study, presence and accuracy of each component of the discharge summary are reported as proportions and 95 percent confidence intervals (CIs). For some hospitalizations, certain items of the discharge summary were not relevant (e.g., “reason for medication changes” if no changes had been made), and in such cases were excluded from the denominator, i.e., were not counted as missing. The target sample size for this component of the study was calculated based on a desired 95% CI for the proportion of charts for which a given item was present/absent of +/− 0.1, using an expected proportion of 0.5 since 95% CIs are widest for proportions of 0.5 (i.e. a conservative estimate). Chance-corrected inter-rater agreement regarding the accuracy of each component of the discharge summary was assessed using the kappa statistic [14].

For the second part of the study, Likert scale responses were grouped into the following categories for analysis: for information regarding most responsible diagnosis and course in hospital, 1 or 2 = inadequate, 3 = ideal, 4 or 5 = excessive; for follow-up plan for medications, further investigations, and consultant/specialist physicians: 1 or 3 = insufficient, 2 or 4 = good. The survey response data are reported as proportions.

The study was approved by the Hamilton Health Sciences Research Ethics Board and the St. Joseph's Healthcare Hamilton Research Ethics Board.

## Results

### Completeness and accuracy of PGY-1 discharge summaries

Ninety discharge summaries dictated by n = 24 McMaster PGY-1 residents during their first two months (July-August 2009) on the Clinical Teaching Units (30 discharge summaries from each of the three university-affiliated teaching hospitals) were independently assessed in duplicate for completeness and accuracy. Most items were completely reported with a given item missing in 5% of summaries or fewer, with the exception of “reason for medication changes” which was missing for approximately 1 in every 6 summaries (Table 1). Inter-rater agreement regarding the accuracy of items was substantial. Approximately 1 in every 3 discharge summaries had inaccuracies for medication related items (medication list, medication changes, and reason for changes) when compared to the patient chart as a reference standard (Table 2).

**Table 1:** Frequency of missing information in discharge summaries

	Missing n/N*	**% (95% CI)**
Discharge diagnosis	1/90	1.1 (0.0-6.6)
Discharge medication list	5/89	5.6 (2.1-12.8)
Medication change(s)	4/82	4.9 (1.5-12.3)
Reason for medication change(s)	13/82	15.9 (9.5-25.4)
Clinical course in hospital	0/90	0.0 (0.0-4.9)
Result of relevant investigations	3/90	3.3 (0.7-9.8)
Follow-up instructions for family physicians	3/87	3.4 (0.7-10.1)

Abbreviations: 95 % CI, 95 % confidence interval.

*Note that some discharge summaries in the study sample are excluded from the denominator if a given item was not applicable for the patient’s stay in hospital (e.g., “Reason for medication change(s)” would not be relevant for a patient whose medications were not changed during that hospitalization).

**Table 2:** Frequency of inaccurate information in discharge summaries, when information was present

	Inaccurate n/N*	**% (95% CI)**	**Inter-rater reliability (kappa)**
Discharge diagnosis	4/89	4.5 (1.4-11.4)	0.79
Discharge medication list	30/84	35.7 (26.3-46.4)	0.94
Medication change(s)	23/78	29.5 (20.5-40.4)	0.75
Reason for medication change(s)	26/69	37.7 (27.2-49.5)	0.79
Clinical course in hospital	12/90	13.3 (7.6-22.0)	0.63
Result of relevant investigations	7/87	8.0 (3.7-15.9)	0.46
Follow-up instructions for family physicians	15/84	17.9 (11.0-27.5)	0.83

Abbreviations: 95 % CI, 95 % confidence interval.

*Note that some discharge summaries are excluded in the denominator, either because the given item was not relevant to the patient’s stay in hospital, or because information for a given item (e.g., discharge medication list) was simply missing, since accuracy could only be assessed if information was present (and relevant to the hospital stay).

Sixty-three (70%) of the discharge summaries were dictated within 48 hours of patient discharge. Carbon copies were sent to a complete list of involved physicians in 75 (88.3%) cases. When rated on overall brevity and completeness, 46 (51.1%) summaries were rated as concise and complete, 28 (31.1%) were rated as long and complete, 9 (10.0%) were rated as long with significant omissions, and 7 (7.8%) were rated as brief with significant omissions.

### Family physicians’ assessment of PGY-1 discharge summaries

Twenty-one family physicians participated in reviewing a total of 68 PGY-1 discharge summaries regarding their own patients discharged from the CTU between July 2009 and February 2010 (Table 3). Surveys were returned for 68/72 discharge summaries, corresponding to a response rate of 94%. Of the items family physicians were asked to rate, communication of post-discharge follow-up plans for investigations was the most frequently identified area for improvement (27.7% of summaries rated as insufficient).

**Table 3:** Family physicians' assessment of PGY-1 discharge summaries

**Item**	**n (%)**
Information regarding most responsible diagnosis	
Inadequate	3 (4.7)
Ideal	51 (79.7)
Excessive	10 (15.6)
Information regarding course in hospital	
Inadequate	6 (9.2)
Ideal	49 (75.4)
Excessive	10 (15.4)
Follow-up plan for medications	
Insufficient	8 (12.3)
Good	57 (87.7)
Follow-up plan for further investigations	
Insufficient	18 (27.7)
Good	47 (72.3)
Follow-up plan with consulting/specialist physicians	
Insufficient	10 (15.4)
Good	55 (84.6)
Length of discharge summary	
Too short	4 (6.3)
Ideal	51 (81.0)
Too long	8 (12.7)

All data are presented as number (percent).

Family physicians also provided qualitative comments regarding areas for improvement in the discharge summary that would have facilitated post-hospital care of the patient. The comments were grouped into the following categories, with the frequency of comments in each category listed in parentheses:

1) Need for a timely discharge summary (n = 13), e.g. “received Aug 12 with discharge July 16 – useless!!”, “needs a timely delivery”

2) Need for clarity regarding follow-up plan (medications, investigations, etc.) (n = 7), e.g. “whenever it is recommended that a follow-up test be done, please state who is to arrange this investigation”, “follow-up plan needs to say what needs to happen, who is expected to make it happen, and what has already been arranged”

3) Need for clarity regarding details of course in hospital (n = 6), e.g. “needs lytes and creatinine on admission recorded”, “didn't include results of investigations”

4) Length of discharge summary (n = 5), e.g. “too long”, “could be a little more concise”

## Discussion

This study of PGY-1 Internal Medicine residents’ discharge summaries found that medication details were frequently omitted or inaccurate, and that family physicians identified lack of clarity about follow-up plans regarding further investigations and visits to other consultants as the areas requiring the most improvement.

Our study findings extend current knowledge regarding the completeness and accuracy of discharge summaries in several ways. First, previous studies have examined primarily the presence or absence of key components of the discharge summaries [11]. In addition to the presence of absence of key items, our study also assessed, when present, the accuracy of these items compared to information contained in the hospital chart, giving a more complete assessment of the quality of the discharge summary. Second, we elicited direct feedback from family physicians about specific discharge summaries they received regarding their own patients after hospital discharge. Most other studies have reported the results of questionnaires that asked physicians to draw on their recollection of recent experience for global feedback about what components of discharge summaries, in general, they find most important and most lacking [15-19]. One study did elicit UK general practitioners’ feedback about specific discharge summaries they received regarding their own patients discharged from hospital and found that 20% of respondents were dissatisfied with the content although the investigators did not quantify the nature of the deficiencies [20]. In another study, a postal questionnaire was administered to general practitioners in the UK regarding specific patients discharged from a variety of inpatient settings (general medicine, general surgery, otolaryngology, geriatrics, gynaecology, ophthalmology, radiotherapy, trauma/orthopedics) and found that information was inadequate with respect to follow-up plans (23% of summaries) and medications at discharge (9% of summaries) [21]. Thus, our study provides further empirical data at a patient-specific level during “real-life” application about components of the discharge summary needing improvement. Finally, our study is novel because it highlights the deficiencies contained within discharge summaries specifically produced by junior housestaff, therefore identifying important priorities for a future educational intervention directed at junior learners.

Our study had several strengths. Assessments of the accuracy of discharge summaries were done using a standardized, pre-piloted form, and showed good inter-rater reliability. The family physician survey response rate was excellent at 94%, and therefore minimizing threats to validity (i.e., responder bias) that can arise from low response rates. Our study also has limitations. First, we assessed accuracy of the discharge summaries using the patient chart as the gold standard and it is possible that the medical record itself was inaccurate. A much more resource-intensive prospective study design would have been required to address this limitation and would not have been feasible for our project. Second, our sample was limited to internal medicine PGY-1 residents and thus the results are not necessarily generalizable to different PGY levels or specialties. Third, investigators performing the assessments of discharge summary completeness and accuracy were not blinded to the identity of the PGY-1 residents who authored the summaries. This may have biased the assessments, since the assessors were senior medical residents who may have worked clinically with some of the PGY-1 residents who authored the discharge summaries and, as a result, may have had preconceived notions about the clinical skills of particular PGY-1 residents. However, the assessment form was intentionally designed to be as objective as possible and we used independent duplicate assessments of the discharge summaries to minimize the potential for bias.

The results from the quality assessment highlight areas for improvement in PGY-1 discharge summaries, specifically the accurate reporting of current and modified medications, along with the rationale for changes made. Adverse drug events account for 72% of all adverse events post-discharge, and half of these are preventable [4]. Of the discharge summaries in our sample for which a discharge medication list was present, almost 40% contained inaccuracies in the list of discharge medications. This error rate is higher than in previous studies (which found error rates of 4.1% and 14.1%), which may reflect our exclusive focus on junior housestaff (incoming first year residents) and perhaps the more frequent and often multiple handovers of patient care during a single hospitalization that occur today as compared to 10 to 20 years ago when the prior studies were conducted [10,22]. Furthermore almost 30% of the summaries in our study did not list medication changes in hospital, and of those that did, many did not explicitly state the reason for the medication change. Awareness about the importance of medication reconciliation is increasing and improvements in this area have been identified as a national patient safety goal by the Joint Commission on Accreditation of Healthcare Organizations [8].

Our study also identified the need to more clearly communicate the details of further or pending investigations and for appointments with specialist physicians. Approximately 40% of patients are discharged from hospital with pending investigations. Nine percent of these investigations ultimately require a change in the medical management of a patient [23]. Thus it is concerning that we found nearly 20% of follow-up instructions regarding investigations were inaccurate. Moreover, the family physicians often commented that it was unclear who was to arrange any necessary follow-up investigations.

The family physicians also commented on several discharge summaries that had not arrived in time for their first post-hospital visit with the patient, rendering the summary significantly less useful and leaving room for medication error. Delayed or incomplete discharge summaries have been shown to affect follow-up management in almost one quarter of cases [20]. In our system, two of the affiliated teaching hospitals (part of a single corporation) encourage physicians to dictate all discharge summaries within 48 hours of patient discharge. In our study, 30% of the dictated summaries were dictated later than 48 hours after discharge, with a range that spanned to 28 days post discharge. Reinforcement of this goal with an explanation of the rationale and the potential implications of delays on patient care could also be reinforced to the residents through an educational initiative.

Currently there is no standard curriculum for incoming medical residents at our institution or for students enrolled across all Canadian medical schools to learn how to prepare a discharge summary. Teaching is informally administered by more senior residents or staff physicians, and thus there is likely wide variability in what information residents would have received prior to our study. Our study identifies information gaps in PGY-1 discharge summaries and could inform the design of an educational intervention to improve the accuracy of residents’ discharge summaries and the relevance of the summaries to ambulatory care providers. Myers et al. described a successful educational initiative using didactic teaching and individualized feedback that could form the basis for our curriculum, with tailoring of the intervention for our institution to address the most prominent gaps identified in our study [24]. The authors envision such an educational intervention as consisting of multiple parts, including a didactic information session outlining the key components of a complete discharge summary, the importance of an accurate and timely discharge summary in providing safe, high quality care, and the most relevant components of the summary to the receiving physician providing post-discharge care in the ambulatory setting (e.g., provision of specific details regarding follow-up care). Small group sessions involving critical review and discussion of examples of desirable and undesirable discharge summaries would be designed to reinforce concepts covered during the didactic session. Periodic evaluation of discharge summaries by the residents' attending physicians would be an important longitudinal component of the educational initiative.

## Conclusions

Discharge summaries are an essential component of transition from inpatient to outpatient care. Our study has outlined pertinent areas for improvement, particularly in areas of medication reconciliation and in the communication of follow-up plans. Our findings will aid in the development of educational interventions for residents.

## Abbreviations

CI: Confidence interval; CTU: Clinical teaching unit; PGY-1: Post-graduate year 1.

## Competing interests

The authors have no conflicts of interest to declare.

## Authors’ contributions

All authors listed have contributed sufficiently to the project to be included as authors; specific contributions are as follows: Dr. Kimberly Legault – study design, data collection, data analysis, manuscript drafting, manuscript editing, final approval of manuscript to be published. Dr. Jacqueline Ostro – data collection, data analysis, manuscript drafting, manuscript editing, final approval of manuscript to be published. Dr. Zahira Khalid – study design, data collection, manuscript editing, final approval of manuscript to be published. Dr. Parveen Wasi – study design, manuscript editing, final approval of manuscript to be published. Dr. John You – study design, data analysis, manuscript editing, final approval of manuscript to be published.

## Pre-publication history

The pre-publication history for this paper can be accessed here:

http://www.biomedcentral.com/1472-6920/12/77/prepub

## Supplementary Material

Additional file 1 Discharge Summary Quality Assessment Form. Click here for file

Additional file 2 Assessment Form for Dictated Discharge Summary – Family Physician. Click here for file
