# Supplementary material for: Quality of discharge summaries prepared by first year internal medicine residents
Source: BMC Med Educ. 2012 Aug 15;12:77. doi: 10.1186/1472-6920-12-77 (PMC3532338; doi:10.1186/1472-6920-12-77)
Supplement: Additional file 1 — Discharge Summary Quality Assessment Form. [file 1472-6920-12-77-S1.doc]

Appendix 1

Discharge Summary Quality Assessment Form

Code number:

Discharge diagnosis (check the appropriate boxes)

□ Present

□ Not present

□ Accurate

□ Inaccurate

Comment on features of diagnoses designated not present or not accurate: ______________________________________________________

List of discharge medications (including doses)

□ Present

□ Not present

□ Accurate (as compared to discharge medication record)

□ Inaccurate (as compared to discharge medication record)

Comment on number of medications designated not accurate or not present: ______________________________________________________

Changes in medications (change in doses, discontinuation)

□ Present

□ Not present

□ Accurate

□ Inaccurate

□ Not applicable

Comment on number of medication changes designated not accurate or not present: ______________________________________________________

Reason for medication changes

□ Present

□ Not present

□ Accurate

□ Inaccurate

□ Not applicable

Comment on number of medication changes for which reason for change is lacking: ______________________________________________________

Clinical course in hospital (mention of all relevant issues resulting in morbidity, affecting length of stay in hospital and/or impacting follow-up)

□ Present

□ Not present

□ Accurate

□ Inaccurate

Comment on relevant aspects of the clinical course that were not mentioned: ______________________________________________________

Relevant investigations and their results (results that had a significant impact on the patient’s clinical course - eg. positive troponins, change in renal/liver function, positive CXR and ECG findings, positive blood cultures, etc.)

□ Present

□ Not present

□ Accurate

□ Inaccurate

Comment on relevant investigations that were missing or incomplete: ______________________________________________________

1. Follow-up instructions for physicians involved in patient care after discharge

□ Present

□ Not present

□ Accurate

□ Inaccurate

Comment on how follow-up instructions differed from those mentioned in the chart: ______________________________________________________

Carbon copy specified to be sent to all physicians involved in the care of the patient

□ Present

□ Not present

□ Accurate

□ Inaccurate

Comments : ______________________________________________________

Overall Brevity and conciseness of the discharge summary

□ Brief with significant omissions

□ Concise and complete

□ Long with significant omissions

□ Long and complete

Length of discharge summary (number of pages): ______________

Timeliness in dictating a discharge summary (within 48 hours of discharge)

□ Dictated less than 48 hours after discharge

□ Dictated more than 48 hours after discharge

Length of time elapsed between discharge and discharge dictation (days): ___________
