# Supplementary material for: Quality of discharge summaries prepared by first year internal medicine residents
Source: BMC Med Educ. 2012 Aug 15;12:77. doi: 10.1186/1472-6920-12-77 (PMC3532338; doi:10.1186/1472-6920-12-77)
Supplement: Additional file 2 — Assessment Form for Dictated Discharge Summary – Family Physician. [file 1472-6920-12-77-S2.doc]

Appendix 2

Assessment Form for Dictated Discharge Summary – Family Physician

Dictation number: Patient’s Initials:

Demographics – Family Physician:

Gender: M F Number of years in practice: _______________

Information regarding diagnosis (ie the main reason for the patient’s hospital stay):

Insufficient Ideal Excessive (Omitted, vague) (much unimportant detail)

1-----------------------2-----------------------3-----------------------4-----------------------5

Comments:

Information regarding the patient’s course in hospital:

Insufficient Ideal Excessive

(Long stays w/ no explanation, (much unimportant detail)

known omitted events)

1-----------------------2-----------------------3-----------------------4-----------------------5

Comments:

Follow-up plan for medications (plans for further med additions, deletions, dose titrations, etc):

1 – No statement about medication plan

2 – Explicit statement that no medication changes required

3 – Follow-up plan included, but incomplete and/or unclear

4 – Follow-up plan included, complete, and clear

Comments:

Follow-up plan for further investigations (what investigations are pending or required, who is to order this, etc.):

1 – No statement about plan for further investigations

2 – Explicit statement that no follow-up investigations are required

3 – Follow-up plan included, but incomplete and/or unclear

4 – Follow-up plan included, complete, and clear

Comments:

Follow-up plan with consulting/specialist physicians:

1 – No statement about plan for follow-up

2 – Explicit statement that no follow-up is required

3 – Follow-up plan included, but incomplete and/or unclear

4 – Follow-up plan included, complete, and clear

Comments:

Please see over →

Length of discharge summary:

Too short (likely omission of detail) Ideal Excessively long

1-----------------------2-----------------------3-----------------------4-----------------------5

What about this discharge summary could be improved to facilitate your post-hospital care of the patient?

Was this discharge summary available prior to the patient’s first post-hospital visit?

Yes No
